# The Cohesin Complex and Its Interplay with Non-Coding RNAs

**DOI:** 10.3390/ncrna7040067

**Published:** 2021-10-22

**Authors:** Merve Kuru-Schors, Monika Haemmerle, Tony Gutschner

**Affiliations:** 1 Junior Research Group ‘RNA Biology and Pathogenesis’, Faculty of Medicine, Martin Luther University Halle-Wittenberg, 06120 Halle, Germany; merve.kuru@uk-halle.de; 2 Institute of Pathology, Section for Experimental Pathology, Faculty of Medicine, Martin Luther University Halle-Wittenberg, 06120 Halle, Germany; monika.haemmerle@uk-halle.de

**Keywords:** cancer, cohesion, miRNA, ncRNA, STAG1, STAG2, RAD21

## Abstract

The cohesin complex is a multi-subunit protein complex initially discovered for its role in sister chromatid cohesion. However, cohesin also has several other functions and plays important roles in transcriptional regulation, DNA double strand break repair, and chromosome architecture thereby influencing gene expression and development in organisms from yeast to man. While most of these functions rely on protein–protein interactions, post-translational protein, as well as DNA modifications, non-coding RNAs are emerging as additional players that facilitate and modulate the function or expression of cohesin and its individual components. This review provides a condensed overview about the architecture as well as the function of the cohesin complex and highlights its multifaceted interplay with both short and long non-coding RNAs.

## 1. Introduction

Spatial and temporal regulation of gene expression is a complex process that involves diverse players and has to be orchestrated on multiple layers. Epigenetic and transcriptional regulators are complemented by post-transcriptional and post-translational modulators to facilitate tight control of RNA and protein abundance in cells. While epigenetic enzymes and transcription factors play a major role in gene expression control in the nucleus, the importance of the cohesin complex is becoming increasingly recognized. Next to its canonical role in sister chromatid cohesion, the cohesin complex also functions in transcriptional regulation and DNA repair [1,2]. However, several molecular details regarding the precise function and regulation of the cohesin complex remain to be resolved. Importantly, transcriptional and post-transcriptional regulation by non-coding RNAs (ncRNAs) also contributes to the fine-tuning of gene expression levels. For example, long ncRNAs (lncRNAs), i.e., transcripts longer than 200 nucleotides (nts), can execute a multitude of molecular mechanisms that can include direct RNA–RNA, RNA–DNA, or RNA–protein interactions. These interactions can alter the localization, translation, stability, or splicing of target transcripts, and lncRNAs have been shown to control larger gene expression networks [3,4]. In addition to lncRNAs, microRNAs (miRNAs) are an abundant and conserved class of small (~22 nts) ncRNAs that assemble with Argonaute (Ago) proteins into miRNA-induced RNA silencing complexes (RISCs) to direct post-transcriptional silencing of complementary RNA targets. A single miRNA can silence several hundred transcripts and the majority of human transcripts carry conserved binding sites for multiple miRNAs [5]. Silencing is achieved through a combination of translational repression and mRNA destabilization, with the latter contributing to most of the steady-state repression in animal cells. Degradation of the mRNA target is initiated by deadenylation, which is followed by decapping and 5′-to-3′ exonucleolytic decay [6]. MiRNA-mediated gene expression control is of outmost importance in diverse developmental and physiological processes. Hence, deregulation of miRNA expression is often found in human diseases, e.g., cancer [7,8].

In this review, we aim to shed light on the interplay between ncRNAs and the cohesin complex. First, we will provide an overview about the cohesin complex and its architecture followed by a description of its diverse biological functions. Ultimately, we will present recent insights into the role of ncRNAs in modulating the expression as well as the functions of the cohesin complex.

## 2. Components of the Cohesin Complex

**Figure 1:**
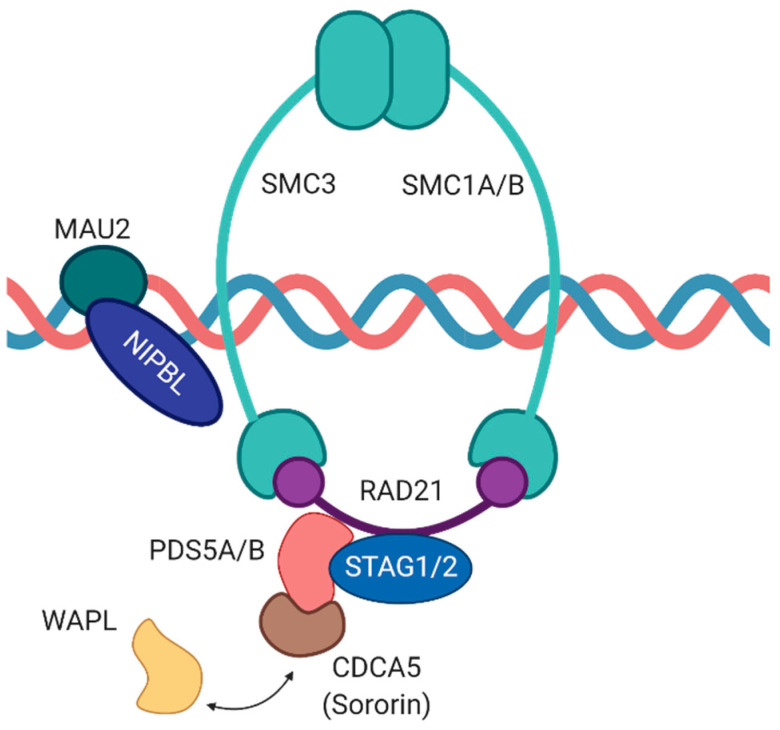
Overview of the cohesin complex and its associated proteins. Four proteins (SMC1, SMC3, RAD21, STAG) constitute the core and form a large ring to encircle DNA strands. The NIPBL/MAU2 heterodimer loads cohesin onto DNA, whereas WAPL/PDS5 release cohesin from chromosomes. CDC5A competes with WAPL for binding to PDS5.

The chromosome-associated cohesin complex is a multi-subunit protein complex which is deeply conserved in eukaryotes with close homologs also found in bacteria. The complex consists of four core subunits as well as several associated proteins (Figure 1). By leveraging the power of genetic screens, several of these components had been initially discovered in yeast and *Drosophila melanogaster* and were shown to be part of the “cohesive” force opposing the “splitting” force exerted by microtubules [9,10,11,12,13,14,15].

Two of the four core subunits of cohesin, namely SMC1A and SMC3, belong to the structural maintenance of chromosomes (SMC) protein family of ATPases. An unusual characteristic of these SMC proteins is their domain organization. In detail, a central hinge domain enables back-folding of the polypeptide chain on itself resulting in an extended anti-parallel coiled-coil structure and the juxtaposing of the N- and C-terminal regions that jointly form a globular ATPase head domain [16,17]. Importantly, SMC1A and SMC3 are tightly connected via their hinge domains within the cohesin complex. Both proteins are joined by Sister chromatid cohesion 1 (Scc1)/RAD21, another core subunit of the cohesin complex, which binds to the ATPase head domains of the SMC proteins thereby closing the tripartite ring-shaped complex [12,18]. Furthermore, Scc1/RAD21 interacts with the fourth core member of the cohesin complex, namely Scc3. In vertebrates, two Scc3 homologs exist, called stromal antigens 1 and 2 (SA1/STAG1 and SA2/STAG2). Of note, both proteins are mutually exclusive core components which stabilize the cohesin complex [19]. STAG2-containing cohesin complexes are generally more abundant in somatic cells and foster binding to the centromere while STAG1-cohesin promotes preferential binding to telomeres [20,21].

Of note, cohesin complexes in meiotic cells slightly differ from their somatic counterparts. While the core complexes are very similar, individual subunits are being exchanged for their meiotic counterparts: instead of SA1/STAG1 or SA2/STAG2 the meiotic complex contains SA3/STAG3, SMC1A is substituted for SMC1B, and RAD21 is replaced by either the meiotic Recombination Protein 8 (REC8) or RAD21-Like protein 1 (RAD21L1) to form the final cohesin ring [22,23].

Next to the four core subunits of the cohesin complex, additional accessory proteins are involved in regulating chromatin binding and dissociation of the ring-shaped multi-protein complex. In dividing cells, Scc2 and Scc4 proteins (Nipped-B-Like protein (NIPBL) and MAU2 in vertebrates) form a heterodimeric cohesin deposition complex which facilitates loading of cohesin onto chromatin during telophase [24]. Importantly, association of cohesin with chromatin is highly dynamic. In vertebrates, precocious dissociation of sisters 5 (PDS5) together with cell division cycle associated 5 (CDCA5), also called Sororin, and Wings apart-like protein homolog (WAPL) form a cohesin-regulatory complex in which Sororin and WAPL compete for binding to the same site on PDS5 thereby affecting the association of cohesin with chromatin either positively or negatively. While the PDS5-Sororin complex maintains sister chromatid cohesion from S to G2 phase, the formation of a PDS5-WAPL complex plays a role in releasing the cohesin complex from chromatin [25,26,27,28].

## 3. Functions of the Cohesin Complex

### 3.1. Sister Chromatid Cohesion

The cohesin complex is best known for its function in mediating sister chromatid cohesion. The complex is loaded onto chromatin after exit from mitosis in telophase or early G1 phase [29]. Loading is facilitated by the NIPBL/MAU2 complex, which promotes ATP hydrolysis by the SMC head domains [30,31,32]. This in turn leads to conformational changes revealing an entry gate at the SMC1/SMC3 hinge where chromatin can enter [33,34]. The association of cohesin with chromatin is kept dynamic through the competing action of the antagonist WAPL, which promotes opening of the ring at the interface between SMC3 head domain and the N-terminus of RAD21 leading to dissociation of the complex [34,35,36]. On chromatin cohesin binds to so-called cohesin attachment regions (CARs) [37,38,39]. There are various models to describe possible binding mechanisms among which the “embrace” model proposes opening of the ring and closing around two strands of DNA [16,40]. Alternatively, the “handcuff” or “snap” model describes how two cohesin complexes each bind a single chromatid and promote cohesion through their dimerization [41]. Lastly, the “bracelet” model suggests an oligomerization of SMC heterodimers to form filaments [41]. Once the cohesin complex is loaded onto chromatin cohesion is established during DNA replication in S-phase of the cell cycle. Acetylation of the lysine residues K105 and K106 in the SMC3 head domains drives cohesion and promotes the interaction of cohesin with Sororin [42]. Sororin then binds to PDS5B thereby stabilizing cohesion and antagonizing WAPL binding. After proper alignment of chromatids at the metaphase plate the prophase pathway is activated for release of cohesin and subsequent initiation of segregation. This is regulated by different mitotic kinases such as Cyclin-dependent kinase 1 (Cdk1) and Aurora B which are involved in phosphorylation of Sororin for its removal from cohesin [43]. Removal of the complex from chromatin further depends on STAG2 phosphorylation via Polo-like kinase 1 (Plk1) [44]. However, some cohesin complexes remain associated with chromatin throughout prophase—mainly at the centromeres. These complexes are protected from the prophase pathway by Shugoshin 1 (Sgo1) [45]. Activation of Sgo1 by Cdk-dependent phosphorylation enables the interaction between protein phosphatase 2A (PP2A) and cohesin. This in turn antagonizes the phosphorylation of Sororin thereby stabilizing cohesin at centromeres and preventing premature segregation of chromosomes [46]. At this stage proper assembly of chromosomes is vital. The spindle assembly checkpoint ensures anaphase entry once chromosomes are aligned in a bi-oriented state. Then the Anaphase Promoting Complex/Cyclosome (APC/C) is activated leading to degradation of numerous proteins including Cyclin B and Securin [47]. Both proteins physically interact and inhibit Separase—a protease responsible for cleaving RAD21 and removing the remaining cohesin rings at centromeres [18,48]. In this final stage, poleward movement of sister chromatids can be executed to generate the new daughter cells.

### 3.2. Role of Cohesin in DNA Damage Response

DNA-damaging events that are triggered by extra- and intracellular agents occur frequently during the cell cycle and can lead to diverse defects including DNA double strand breaks (DSBs). In order to repair DSBs, two major pathways exist in eukaryotes, namely non-homologous end-joining (NHEJ) and homologous recombination (HR). The NHEJ pathway re-joins the ends of damaged DNA, which can result in an imperfect restoration of the genetic information due to the random gain or loss of one or more nucleotides at the DSB site. In contrast, the HR pathway is able to restore the original sequence by utilizing the nearby sister chromatid as a template to faithfully repair the damaged DNA. However, the HR pathway is only active in S- and G2-phase whereas the NHEJ pathway can be used to repair DSBs throughout the cell cycle [49,50]. Given its cohesive function it is not surprising that the cohesin complex plays a highly conserved role in DNA damage response [14,51,52,53]. During DSB repair, cohesin is responsible for checkpoint activation, suppression of transcription at damage sites, and DNA damage-induced cohesion. Upon detection of DSBs by DNA-damage sensor proteins (e.g., ATM, ATR, Mre11/Rad50, NBS1) during S and G2/M phases, cohesin complexes are recruited genome-wide to pre-existing cohesin sites in order to re-enforce cohesion [54]. A specific recruitment of cohesin at sites of DNA strand breaks can occur in a Mre11/Rad50-dependent manner and subsequent phosphorylation of SMC1 and SMC3 by ATM and NBS1 are critical downstream events [55,56,57,58,59]. The DNA damage-induced cohesion facilitates the recruitment of additional checkpoint proteins to damaged DNA sites to activate the respective checkpoints. For example, cohesin has been shown to be required for the intra-S phase as well as G2/M checkpoints by enabling the threonine 68 phosphorylation and thus complete activation of the checkpoint kinase Chk2 after DNA damage-induction in S-, G2-, as well as in G1-phase. Since there is no sister chromatid cohesion in the G1 phase, these findings suggest that the function of cohesin in checkpoint activation and sister chromatid cohesion are independent from each other [60]. Furthermore, recent findings highlight the importance of both cooperativity and division of labor between STAG1- and STAG2-cohesin complexes for genome maintenance and cell survival [61].

In addition to its role in checkpoint activation, cohesin is also required to suppress active transcription at DSB throughout the entire interphase. Importantly, failure to repress transcription near DSBs can lead to large-scale genome rearrangements, such as translocations. However, while SMC3, RAD21, PDS5B, and STAG2 are required for efficient suppression, loss of PDS5A or STAG1 does not affect gene transcription suggesting that PDS5B and STAG2-cohesin complexes have a specific role in transcription suppression at damaged DNA and chromatin regions [62]. Intriguingly, the functional analysis of a cancer-associated STAG2 S202L mutation, which is proficient for sister chromatid cohesion, but not transcriptional repression at DNA breaks suggests that one putative mechanism by which STAG2 functions as a tumor suppressor is through its role in promoting accurate repair at DSBs that occur in the vicinity of actively transcribed genes [62].

Last but not least, cohesin and its subunits were also shown to be involved in NHEJ during immunoglobulin class switch recombination (CSR). CSR is initiated by the transcription-coupled recruitment of activation-induced cytidine deaminase (AID) and the subsequent generation of DSBs. These breaks activate the DNA damage response and are resolved through classical and alternative NHEJ pathways. Importantly, cohesin subunits (SMC1, SMC3, NIPBL, WAPL) were shown to interact with nuclear and chromatin-bound AID and are required for efficient CSR [63].

### 3.3. Function of Cohesin in Chromatin Architecture and Gene Expression

To facilitate chromosome-structure compaction and gene regulation, the genomes of eukaryotic cells are organized into compartments, topologically association domains (TAD), and loops [64,65]. Next to its functions in sister chromatid cohesion and DNA repair, the cohesin complex also organizes interphase chromatin thereby regulating gene expression and chromatin architecture [66]. For example, cohesin affects several steps in RNA Pol II-mediated transcription including initiation, elongation, and termination [2,67,68,69,70]. Furthermore, in association with CCCTC-binding factor (CTCF), cohesin has an architectural role in 3-dimensional chromatin folding, generating CTCF loop domains and bringing cis-acting elements (e.g., enhancers) into the proximity of gene promoters [71]. In detail, the cohesin complex entraps and extrudes DNA to form loops that are restricted by chromatin-bound CTCF [72,73,74,75]. These loops facilitate enhancer–promoter interactions or form insulated hubs thus preventing transcription [71,76,77]. Together with CTCF cohesin is important for the hierarchical organization of DNA loops into TADs [77,78,79,80]. CTCF is a zinc-finger protein that is required for transcriptional insulation. It binds DNA through an 11-zinc-finger domain and can directly interact with STAG2-RAD21 sub-complex through its N-terminal segment [81]. For DNA loop extrusion, CTCF is bound to convergently oriented “loop anchor” DNA sequences at the base of loops, thereby defining the boundaries of TADs and halting extrusion upon encounter of CTCF by cohesin on DNA [74,82]. TADs are further segregated into compartments or can even be part of larger compartments, representing active or inactive chromatin [65,71,73,77,83,84,85]. Cohesin regulatory proteins are also crucial for loop extrusion and chromatin organization [84,85,86,87]. For example, depletion of RAD21 and NIPBL has been shown to strengthen compartmentalization, implying that cohesin mediated loop extrusion counteracts epigenetic compartmentalization. This might be required to prevent the largescale spread of transcriptionally active or inactive states [71,77,84]. Of note, depending on the genomic site and cellular context, cohesin can facilitate as well as antagonize the organization of Polycomb group-associated domains [88,89,90]. This is at least partially explained by the redundant and non-redundant roles of the STAG1 and STAG2 subunits in chromatin organization and gene expression [88,91,92,93,94,95]. Both, STAG1 and STAG2, co-localize with CTCF and have common and independent DNA binding sites. Importantly, STAG1-cohesin was shown to stabilize TAD boundaries and disrupt long-range Polycomb repressive complex 1 (PRC1) interactions, which counteracts compartmentalization. In contrast, STAG2-cohesin complexes facilitate local enhancer–promoter interactions and long-range PRC1 interactions required for gene repression [88,94,95]. Interestingly, STAG1 or STAG2 depletion does not generally affect TADs, suggesting that both subunits can compensate for each other to maintain chromatin architecture [96]. However, STAG1 is unable to compensate for the loss of STAG2 at a subset of STAG2-specific sites in key lineage-defining genes [94,95]. Importantly, mutation of individual subunits of cohesin and CTCF or the complete loss thereof can result in loss of loop extrusion and thereby affect the hierarchical organization of chromatin as well as gene expression regulation [97]. Consequently, loss of cohesin activity can impact normal cellular function and development including proliferation and pluripotency [98,99]. Yet, surprisingly, despite the loss or alterations in TADs upon cohesin depletion, only minor effects on steady-state gene transcription and enhancer activity could be observed [71,100]. However, it has been shown that cohesin is critically required for inducible gene expression and enhancer dynamics suggesting an important role for the cohesin complex in the transition from a resting to an activated cell state. The cohesin-dependence of inducible genes renders inducible gene expression particularly vulnerable to disruption by the loss of cohesin [83,100,101,102].

In summary, the loop extrusion activity of the cohesin complex and its regulation is fundamental for genome organization and constitutes a source of chromatin contacts with important implications for gene expression regulation [2].

## 4. The Cohesin Complex and Its Interplay with Non-Coding RNAs

With the event of large-scale RNA sequencing, both small and long non-coding RNAs have been found to constitute a large portion of the human transcriptome. While our understanding of the molecular role of these transcripts is far from being complete, it is becoming more and more obvious that fundamental questions in biology might only be resolved by considering the action of non-coding RNAs. In fact, several studies over the last decade demonstrated the multidimensional functions of lncRNAs. For example, cytosolic miRNAs and lncRNAs are thought to mainly regulate mRNA decay and translation, protein stability, as well as interfere with each other’s function. In contrast, nuclear ncRNAs have been shown to epigenetically regulate chromatin remodeling and structure as well as gene transcription through their interaction with chromatin modifying enzymes and transcription factors or by adopting an architectural role in the construction of the cell nucleus and maintenance of the three-dimensional organization of the genome [103,104,105]. Some of these nuclear functions of ncRNAs overlap with the aforementioned activities of the cohesin complex and its subunits. Indeed, previous studies revealed an intimate connection between ncRNAs and CTCF that underlie the locus-specific recruitment of CTCF and are implicated in long-range chromosomal interactions [106,107,108]. While these findings highlight the interaction of individual ncRNAs with CTCF to achieve transcriptional and architectural regulation, the regulation of core cohesin subunits by ncRNAs remains largely neglected. For example, expression control of cohesin subunits by ncRNAs is poorly understood. Moreover, the contribution of ncRNAs in mediating or modulating the multitude of cohesin function remains largely unclear. Here, we present recent insights into these questions by systematically introducing the multilayered regulation of cohesin complex expression and function through both small and long non-coding RNAs.

### 4.1. Regulation of the Cohesin Complex Subunits by MicroRNAs

MicroRNAs (miRNAs)—short ~22 nts long non-coding RNAs—are important regulators of post-transcriptional gene silencing. MiRNA genes are transcribed by RNA polymerase II yielding an initial primary transcript, called pri-miRNA, which consists of a stem-loop structure containing the mature miRNA sequence [109]. The pri-miRNA is further trimmed by the Microprocessor consisting of the nuclear RNase III Drosha and its cofactor DiGeorge Syndrome Critical Region Gene 8 (DGCR8) yielding a small hairpin-shaped RNA (pre-miRNA) [110,111]. Following processing by Drosha, the pre-miRNA is exported into the cytoplasm facilitated by the transport complex, which includes Exportin 5 (XPO5) and GTP-binding Ras-related Nuclear Protein RAN [112]. In the cytoplasm, the pre-miRNA is further processed by Dicer—another RNase III-type endonuclease [113,114]. Finally, the resulting small RNA duplex is loaded onto an Ago protein to form the RISC thus allowing the targeting of specific transcripts via a target recognition site at the 5′ end of the miRNA termed the “miRNA seed region” [115]. The short recognition motif needed for target identification allows for the control of a plethora of RNAs by one single miRNA. Once the RISC is directed towards a target transcript via the complementary base pairing between the target and the miRNA, the respective coding or non-coding transcript is subjected to degradation and/or translational inhibition [116,117,118,119]. In human cancers, diverse genetic and non-genetic mechanisms can modulate the expression of mature miRNAs. For example, miRNA genes can be depleted or amplified, their transcription can be altered, or their biogenesis could be affected. Under certain conditions, these miRNAs might act as tumor suppressors or they might promote tumor progression impacting different hallmarks of cancer like proliferation, invasion, migration, and cell death [120,121,122]. Importantly, a deregulation of specific miRNAs or a general alteration of mature miRNA levels could lead to subsequent expression changes of cohesin network components thereby affecting tumor progression. In fact, loss of the endonuclease Dicer in chicken-human hybrid DT40 cells was found to cause disruption of heterochromatin formation and abnormal mitotic cells with premature sister chromatid cohesion and an increase in aneuploidy [123]. The general relevance of the endogenous RNA interference (RNAi) system for proper chromosome architecture and dynamics during mitosis as well as meiosis was also established by early studies in fission yeast. It was shown that the RNAi machinery is required for the accurate segregation of chromosomes and defects were correlated with loss of cohesin at centromeres [124].

Besides these general connections between components of the endogenous RNAi pathway and cohesin, a regulatory impact on cohesin core and accessory subunits has also been assigned to individual miRNAs. For example, a decreased STAG1 expression in colorectal cancers among African Americans has recently been linked to an exonic single nucleotide polymorphism (SNP), namely rs34149860. Intriguingly, the SNP allele contains a binding site for miR-29b and functional inhibition of this miRNA could rescue STAG1 mRNA levels [125]. In addition to STAG1, also STAG2 was recently suggested to be a target of miRNAs. Here, overexpression of miR-22 in human umbilical vein endothelial cells (HUVECs) reduced STAG2 protein levels and identified a putative binding site in the 3′ untranslated region (UTR) of STAG2 mRNA using a luciferase reporter assay [126]. Furthermore, miR-409-5p overexpression and inhibition in a prostate cancer cell line reduced or increased STAG2 mRNA or protein, respectively [127]. Similarly, miR-154* was identified as a regulator of STAG2 in prostate cancer cells by the same research group [128]. Another core cohesin subunit, RAD21, is well-known for its role in drug sensitivity and DNA damage repair signaling. In this context, RAD21 targeting by miR-17 and miR-92 was shown to alter cisplatin resistance [129]. Moreover, the miR-320b/RAD21 axis has been identified as a regulatory mechanism affecting radiosensitivity to ionizing radiation in liver cancer cells [130]. Furthermore, the tumor-suppressive functions of miR-122-5p in cervical cancer are at least partially explained by its inhibitory effect on RAD21 [131].

In addition to the core subunits of the cohesin complex, accessory components of the cohesin network have been shown to be regulated by miRNAs as well. For example, NIPBL was identified as a direct target of miR-187-3p and NIPBL downregulation was shown to facilitate sensitivity towards treatment with propranolol in infantile hemangioma [132]. In non-small cell lung cancer, on the other hand, miR-99b was shown to inhibit invasion and migration at least partially due to its regulatory impact on NIPBL [133]. Furthermore, PDS5B was suggested to be a direct target of miR-27a in human prostate cancer [134]. Importantly, additional members of the cohesin complex were shown to be directly targeted by different miRNAs in diverse cell types, as summarized in Table 1.

These examples highlight an intriguing connection between the RNAi pathway with its role in post-transcriptional gene expression control and the nuclear chromatin structure and function as organized by the cohesin complex.

### 4.2. Role of Long Non-Coding RNAs in Cohesin Functions

Regulation of the cohesin component expression through different miRNAs represents one possible way to control cohesin-dependent effects in cells. However, various other non-coding RNAs are expressed in cells and could modulate the function of the cohesin complex as well. In particular, lncRNAs are implicated in various biological processes such as proliferation, gene expression regulation, and apoptosis. They achieve transcriptional control through interaction with DNA, RNA, and proteins and have the ability to alter chromosomal architecture [145]. Due to their numerous functional roles, lncRNAs take a major regulatory part in a broad range of hereditary and sporadic diseases including developmental disorder syndromes, diabetes, acute injuries, and cancers [3,146,147,148,149]. For example, the lncRNA mouse maternal expressed gene 3 (MEG3) was shown to play a role in insulin biosynthesis in pancreatic islets. In detail, MEG3 can associate with Enhance of zeste homolog 2 (EZH2), a methyltransferase of the PRC2 complex, thereby recruiting this enzyme to the RAD21, SMC3, and SIN3A gene loci and inducing H3K27 methylation leading to epigenetic gene silencing. The inhibition of these genes by Meg3 in turn promotes the expression of v-Maf musculoaponeurotic fibrosarcoma oncogene family, protein A (MafA), and increases insulin synthesis and secretion through MafA [150]. Another example of expression regulation of cohesin subunits by lncRNAs was recently discovered in the context of acute spinal cord injury-associated apoptosis. Here, overexpression of the myocardial infarction-related transcript (MIAT) was found to have a neuroprotective function [151]. Mechanistically, MIAT was shown to bind to the RAD21 protein and inhibit its degradation thereby promoting the RAD21-dependent transcriptional activation of the vascular endothelial growth factor A (VEGFA) gene to promote neuronal cell survival [151]. Besides these RNA-dependent functions of lncRNAs, transcription of a lncRNA gene locus alone can have a regulatory role as well [152]. In line with this, a recent study identified NIPBL-AS1, a lncRNA transcribed upstream and antisense to the NIPBL gene, whose active transcription leads to reduced NIPBL expression levels as shown by knockdown and transcription blocking experiments. Vice versa, blockage of the transcription of NIPBL increased NIPBL-AS1 levels [153]. This regulatory connection is of clinical relevance and blockage of NIPBL-AS1 transcription could alleviate symptoms associated with the Cornelia de Lange syndrome (CdLS). This syndrome is characterized by craniofacial anomalies, upper limb malformations, growth and mental retardation, hirsutism, and other system abnormalities and severely affected patients showed only ~65% whereas mildly affected patients expressed ~75% of normal NIPBL transcript amounts [154,155]. These examples highlight the diverse mechanisms operated by lncRNAs in order to modulate expression of cohesin subunits. However, lncRNAs can also assist the cohesin complex in the formation of intrachromosomal interactions. Here, Oct4 promoter-interacting long noncoding RNA 16 (Oplr16) was recently shown to utilize its 3′-fragment to recruit the chromatin factor SMC1 to orchestrate pluripotency-associated intrachromosomal looping and knockdown of Oplr16 abolished the 3D chromatin structures required for the maintenance of pluripotency [156].

### 4.3. Role of Enhancer RNAs in Cohesin Function

Over a decade ago, enhancer regions were shown to support transcription and give rise to a special class of non-coding transcripts called enhancer RNAs (eRNAs) and there have been numerous reports about cell-type and signal-dependent expression of eRNAs [157,158,159,160,161]. Of note, more than 40,000 eRNAs have been identified in human cells using nascent RNA sequencing approaches that allow annotation of these unstable transcripts [162,163]. While eRNAs have become a hallmark of active enhancers, it remains to be resolved whether enhancer transcription, eRNAs themselves, or both, are important for enhancer activity [164]. For example, enhancer transcription was shown to be important for maintaining an open chromatin state that is accessible for transcription factors [165]. Additional studies have shown that a subset of eRNAs are required for the expression of certain target genes [160]. Thus, eRNA have been shown to perform important functions yet it remains to be determined whether eRNAs act *in cis* versus *in trans*. Given the short half-lives of the majority of eRNAs, eRNAs are generally expected to act *in cis* [157,158,162,165]. However, eRNAs have also been found to locate to distinct chromosomal regions to perform functions *in trans*. An example of an eRNA acting in *trans* was also recently identified in mice [166]. The ^DDR^eRNA originates from an enhancer region of the myogenic master regulator Myoblast Determination Protein 1 (MYOD1) and interacts with different subunits of the cohesin complex, namely SMC1A, SMC3, NIPBL, and PDS5B, to ensure spatially appropriate cohesin loading in *trans* to regulate expression of Myogenin thereby controlling myogenic differentiation. The *trans*-activating function and SMC3 binding ability of ^DDR^eRNA was shown to be implemented through two of a total of four domains that were identified via in silico RNA secondary structure prediction [166]. Of note, SMC3 as well as RAD21 have been shown to interact with several other eRNAs and knockdown of specific eRNAs resulted in a decrease of cohesin recruitment to specific enhancers in response to estrogen stimulation in breast cancer cells [160]. Furthermore, eRNAs were shown to bind to Mediator, a component involved in chromatin looping together with cohesin, and promote its association with NIPBL to stabilize loop formation and reinforce transcription [167,168,169]. In summary, enhancer RNAs can support and modulate the function of the cohesin complex in gene expression regulation, especially through chromatin loop formation and facilitating enhancer–promoter interactions.

### 4.4. Interplay between Different Non-Coding RNA Classes and the Cohesin Network

While certain single non-coding RNAs can directly or indirectly influence their targets, there are known interactions between different kinds of ncRNAs which affect each other’s regulatory functions. For example, lncRNAs as well as circular RNAs (circRNAs) can serve as miRNA sponges. These sponge RNAs contain complementary binding sites for specific miRNAs which allows them to sequester these small inhibitory RNAs thereby preventing the silencing of miRNA targets. An example of this competition for miRNA binding was revealed in Autism Spectrum Disorder (ASD) patient samples. Here, RNA-Seq data of ASD cortex samples were analyzed for circRNA deregulation using available circRNA detection tools. A circRNA of interest, namely circARID1A, was identified through correlation analysis of the circRNA–miRNA–mRNA axis with miR-204-3p as its predicted interactor. Knockdown or overexpression of circARID1A both had no effect on expression of its collinear counterpart (ARID1A), while miR-204-3p was significantly increased or decreased, respectively. Further analysis of ASD implicated genes revealed the downregulation of STAG1 by miR-204-3p overexpression and circARID1A knockdown [140]. Taken together, these results revealed that circARID1A could regulate genes implicated in ASD, including STAG1, through directly sponging miR-204-3p. However, the impact of this circRNA–miRNA–mRNA axis on disease onset and progression needs to be investigated in more detail. Another example of a multi-layered regulation was recently described for CDCA5/Sororin in nasopharyngeal carcinoma (NPC) and hepatocellular carcinoma (HCC) [142,144]. In detail, the lncRNA LINC01515 was shown to be upregulated in NPC and its knockdown decreased cell proliferation, migration, and invasion suggesting an oncogenic role of this lncRNA. Further analysis of the underlying molecular mechanism revealed that LINC01515 could act as a sponge for miR-325 which in turn increases CDCA5 expression by preventing it from being directly targeted by miR-325. Of note, the anti-tumor effects induced by LINC01515 depletion could be partially reversed by CDCA5 overexpression [142]. Similarly, the lncRNA RHPN1-AS1 was found to contribute to liver cancer progression via the miR-485/CDCA5 axis. In particular, RHPN1-AS1 was found to be upregulated in HCC through the activation of STAT1. Functional studies revealed a positive impact of this lncRNA on cell proliferation and motility. Mechanistically, RHPN1-AS1 was suggested to modulate CDCA5 expression via targeting miR-485 [144]. Taken together, these findings suggest that therapeutic delivery of miR-325 or miR-485 could be an effective strategy to interfere with the function of the cohesin complex in NPC and HCC, respectively. However, several open questions remain, and much more rigorous studies are needed in order to deliver a quantitative assessment of these higher-order regulatory networks.

## 5. Conclusions

The cohesin complex and its cohesion-mediating network portray a highly interesting yet incompletely understood mode of regulation that is crucial to any cell. With its binding to chromatin, this ring-shaped complex collectively influences critical pathways and biological processes such as mitotic segregation, DNA damage repair, and transcriptional regulation allowing inter-chromosomal interactions. Furthermore, combining this knowledge with the modulatory function of non-coding RNAs which were shown to interact with cohesin through diverse mechanisms creates an intricate network. In addition to the aforementioned classes of non-coding RNAs, there are many more such as unusually small RNAs (usRNAs) that are also involved in regulation of the cohesin subunits [170]. However, our understanding of the molecular mechanisms of these transcripts is very limited and more detailed investigations are needed to fully understand the intertwined regulatory cues between the cohesin complex and ncRNAs. Research endeavors in this direction are highly warranted given the fundamental roles of cohesin subunits and ncRNAs in several human diseases including cancer [3,83,120,171,172,173]. These efforts will not only broaden our understanding of cohesin and ncRNA biology but might also lead to the development of novel therapeutic strategies.

## Figures and Tables

**Table 1:** Experimentally confirmed miRNAs targeting components of the cohesin complex and additional accessory proteins involved in cohesion establishment.

Gene	miRNAs	Cell Type/Tissue	References
SMC1A	miR-9	Glioblastoma	[135]
	miR-23a-3p	Acute myeloid leukemia	[136]
	miR-139-5p	Prostate cancer	[137]
	miR-215-5p	Acute myeloid leukemia	[138]
	miR-638	Leukemia	[139]
RAD21	miR-17	Lung cancer	[129]
	miR-92	Lung cancer	[129]
	miR-122a-5p	Cervical cancer	[131]
STAG1	miR-29b	Colon cancer	[125]
	miR-204-3p	Human neuronal cells	[140]
STAG2	miR-22	Human umbilical vein endothelial cells	[126]
	miR-409-5p	Prostate cancer	[127]
	miR-154-3p	Prostate cancer	[128]
NIPBL	miR-99b	Lung cancer	[133]
	miR-187-3p	Hemangioma-derived stem cells	[132]
PDS5B	miR-27a	Prostate cancer	[134]
	miR-223	Pancreatic cancer	[141]
CDCA5	miR-325	Nasopharyngeal carcinoma	[142]
	miR-326	Ovarian cancer	[143]
	miR-485	Hepatocellular carcinoma	[144]
